# Redesigning Recruitment and Engagement Strategies for Virtual Culinary Medicine and Medical Nutrition Interventions in a Randomized Trial of Patients with Uncontrolled Type 2 Diabetes

**DOI:** 10.3390/nu15194124

**Published:** 2023-09-25

**Authors:** Molly F. McGuire, Patricia M. Chen, Carolyn Smith-Morris, Jaclyn Albin, Milette D. Siler, Miguel Angel Lopez, Sandi L. Pruitt, Vincent C. Merrill, Michael E. Bowen

**Affiliations:** 1 University of Texas Southwestern Medical Center, Division of General Internal Medicine, Dallas, TX 75390, USAmichael.bowen@utsouthwestern.edu (M.E.B.); 2 Peter O’Donnell Jr. School of Public Health, Dallas, TX 75390, USA; 3 University of Texas Southwestern Medical Center, Division of Combined Internal Medicine and Pediatrics, Dallas, TX 75390, USA; 4 Moncrief Cancer Institute, Fort Worth, TX 76104, USA; 5 Gretchen Swanson Center for Nutrition, Omaha, NE 68154, USA; 6 Harold C. Simmons Comprehensive Cancer Center, University of Texas Southwestern Medical Center, Dallas, TX 75235, USA

**Keywords:** culinary medicine, pragmatic, trial engagement, food insecure, Type 2 Diabetes

## Abstract

In-person culinary medicine (CM) can improve health behaviors, but its translation to virtual platforms and impact on diabetes outcomes are not well described. We designed a pragmatic trial comparing the effectiveness of virtual CM (eCM) to Medical Nutrition Therapy on diabetes outcomes among patients with uncontrolled diabetes within a safety-net healthcare system. All participants were provided cooking equipment and food from a food pantry. Due to low initial eCM participation, recruitment was paused, and eight semi-structured interviews were conducted to solicit feedback on study appeal, operations, and barriers to participation. Rapid thematic analysis was used to modify study operations. We found that participants were interested in the study and motivated by health concerns. While they valued food distribution and cooking equipment, they highlighted transportation barriers and conflicts with the pick-up time/location. Some eCM participants expressed discomfort with the virtual platform or preferred to observe rather than cook along. Study operations were modified by (1) moving supply pick-up to a familiar community clinic and diversifying food pick-up locations; (2) offering an in-person orientation to the program to increase comfort with the virtual platform; (3) emphasizing the credibility and relatability of the eCM instructor and encouraging participation of family members. This redesign led to the recruitment of 79 participants, of whom 75% attended at least one class. In conclusion, participant feedback informed pragmatic changes in study operations that increased engagement in this ongoing trial and may inform future eCM program design.

## 1. Introduction

People with Type 2 Diabetes (T2D) face daily challenges in diabetes self-management, including healthy eating, being active, glucose monitoring, and medication management [1]. Nutrition therapy is foundational to diabetes prevention and management [2]. Medical Nutrition Therapy (MNT) is an evidence-based approach to nutrition therapy that includes diagnostic, therapeutic, and counseling services [3]. Multiple studies have shown that MNT can lower hemoglobin A1c (HbA1c), blood pressure, BMI, waist circumference, and cholesterol [4,5]. Clinical practice guidelines for diabetes recommend referrals to Registered Dietitians (RDs) with specific MNT knowledge; such individualized sessions are demonstrated to positively impact institutional cost savings and cardiometabolic outcomes [2,6,7]. However, the cost to the patient of eating nourishing food [8], the temptation to consume food that does not promote health [9], as well as costs associated with MNT outpatient visits may limit engagement with MNT and be a barrier to improved diabetes outcomes [3].

Culinary Medicine delivered using an RD offers an alternative approach to MNT that provides both nutrition education and experiential cooking. By learning nutritional principles in a “hands-on” manner, participants can improve diet variety and healthy cooking practices and build confidence toward food-related behavior change and kitchen self-efficacy [10,11,12,13]. Culinary medicine has been widely evaluated in academic settings as part of medical trainee curricula [14,15,16,17]. In a community-based trial setting, 6 weeks of culinary medicine improved adherence to the Mediterranean diet and provided cost savings to participants compared with those receiving nutrition counseling from their healthcare providers [18]. When paired with produce distribution, culinary medicine education increased fruit and vegetable consumption after seven weekly classes of community-based culinary medicine [19].

While initial studies of culinary medicine have promising findings, randomized studies in food-insecure patients with diabetes [20] and longitudinal follow-up studies are lacking [10]. Early studies have focused on feasibility, patient experience, and behavior change as primary outcomes, with a secondary focus on clinical outcomes. Pilot studies of virtual culinary medicine suggest potential positive impacts on health outcomes and psychosocial coping [21,22]. Although the COVID-19 pandemic drove programs to transition to virtual programming [23], the feasibility, design, and impact of virtual culinary medicine is not well described.

We designed a pragmatic trial comparing the effectiveness of virtual culinary medicine and clinic-based medical nutrition therapy on T2D outcomes. Patients with uncontrolled T2D were recruited from a local safety-net clinic system with high rates of food insecurity among the patient population. This paper describes the initial recruitment and engagement challenges within the randomized trial of virtual culinary medicine and medical nutrition therapy, the implementation of redesign strategies based on participant and stakeholder feedback, and the impact of these changes on recruitment and engagement in an ongoing RCT.

## 2. Materials and Methods

### 2.1. Study Setting

The highest incidence of estimated diabetes prevalence in North Texas is concentrated in South Dallas and is likely exacerbated by food insecurity [20]. In South Dallas, 27% of the residents live in food deserts, and 40% of households participate in the Supplemental Nutrition Assistance Program (SNAP) [24]. Two South Dallas ZIP codes, 75215 and 75210, experience some of the highest estimated diabetes prevalence in Dallas County (20.4% and 19.7%, respectively) [25]. People living in these areas also have access to Parkland Health’s (“Parkland”) integrated, safety-net healthcare system that primarily serves under- and uninsured populations with comprehensive patient services, including nutrition education. Parkland’s nutrition program at its South Dallas community-oriented primary care clinic (COPC) [26] receives over 1500 system-wide referrals for nutrition services annually. Diabetes management and access to quality food is a priority for Parkland, which has a strong community presence in South Dallas.

### 2.2. Study Design

We designed a pragmatic randomized controlled trial (RCT) to compare the effectiveness of hands-on, experiential cooking classes (culinary medicine intervention) and standard of care, clinic-based medical nutrition therapy for patients with uncontrolled diabetes. This study was approved by the UT Southwestern Medical Center Institutional Review Board (STU 200-1244). Patients were enrolled in the trial for 6 months of active intervention, and an additional 6 months of post-intervention follow-up to evaluate for sustained behavioral and biometric changes. All participants received kitchen utensils and equipment and information about local food assistance. In its original design, we proposed in-person enrollment and cooking classes at a local food pantry and teaching kitchen in a community center within 2 miles of the clinic. However, the COVID-19 pandemic necessitated a shift to virtual visits where culinary medicine classes were delivered via Zoom Cloud Meetings, v5.13.11 [27], an online audio and video sharing platform. This change occurred prior to study launch, and we adapted study procedures to address anticipated challenges with the virtual format.

### 2.3. Study Eligibility

Patients were identified from Parkland’s Type 2 Diabetes Registry, which captures patients using a combination of ICD codes and laboratory data. Those with an assigned primary care provider at the Parkland COPC clinic in South Dallas were eligible if they (1) had a primary care visit in the past 12 months, (2) were ages 18 years or older, (3) had a duration of diabetes > 12 months, and (4) had an HbA1c ≥ 7.0% in the past 3 months. Patients who completed a nutrition visit in the past 12 months, did not speak English or Spanish, had an eGFR ≤ 30, or were on dialysis were excluded. We initially planned to identify eligible patients after completion of the Healthy Living with Diabetes Program, Parkland’s American Diabetes Association-recognized diabetes self-management education program [6]. Due to the COVID-19 pandemic, that program was paused, so we used the existing Type 2 Diabetes Registry in Parkland’s Electronic Health Record (EHR) to identify and recruit eligible patients. We used REDCap v13.6.1 (Research Electronic Data Capture), a secure, web-based software platform designed to support data capture for research studies [28,29] to manage, track, and recruit eligible patients.

### 2.4. Recruitment Strategy

Eligible patients were sent a study invitation letter and information sheet in their preferred language (English or Spanish). A research assistant followed up by phone to confirm eligibility and complete recruitment. Due to the shift to virtual classes, a screening question about internet access was added to the inclusion criteria. Interested patients provided verbal informed consent and completed a baseline telephone survey in their preferred language. After we recruited enough patients (i.e., 30–40) to form a class, each language/class grouping was randomized 1:1 to a treatment arm. During the three-year study period, we randomized three class groupings, each exposed to the intervention for 6 months, followed by an additional 6-month follow-up period.

### 2.5. Participant Enrollment and Food Assistance

Participants were notified by phone of their study arm assignment and scheduled to meet with the study team to complete their enrollment process at an established South Dallas community organization with an on-site food pantry and kitchen suitable for culinary medicine classes. With written informed consent to release their name, we also connected participants to food assistance services. Because food pantry clients often lack kitchen utensils and equipment to cook at home [30], we provided basic kitchen equipment (e.g., frying pans, cutting boards, measuring cups, and cutlery) for all participants regardless of study arm. Due to pandemic-related restrictions, we met with participants using a drive-through distancing approach rather than office-based meetings to complete the written informed consent process baseline survey and administer the donated cooking supplies. We collected the following baseline characteristics: sex, age, preferred language, education level, household size, use of food assistance [31], food insecurity [32] in the last 30 days, and HbA1c value. Additional items were collected but will be presented in future outcomes publications.

### 2.6. Development of Medical Nutrition Therapy Arm (MNT)

We partnered with Parkland Clinical Nutrition to deliver the MNT intervention. Participants randomized to MNT were offered a series of six sessions based on the American Diabetes Association (ADA) recommended Medical Nutrition guidelines for diabetes education [6]. The curriculum included visual cues and handouts, as well as education tailored to each patient’s health status and glucose data. Registered dietitians (RDs) conducted individual and group sessions in the participants’ preferred language (English or Spanish). RDs contacted and scheduled patients into reserved 1-h appointment slots during clinic hours and rescheduled visits as needed. MNT sessions were available either virtually via phone call (for 1:1), WebEx (for group sessions), or in-person at the clinic as permitted by health system policies during the COVID-19 pandemic. Table 1 provides a detailed comparison of the two study arms with respect to staffing, curriculum design, teaching concept, and logistics.

### 2.7. Development of Electronic Culinary Medicine Arm (eCM)

In partnership with culinary medicine-trained clinicians (JA, MDS), our study team accessed the Diabetes and Carbohydrate training module from the Health Meets Food curriculum [33] via institutional license. Elements of the curriculum, recipe selections, intervention timing, scheduling, and class frequency protocol were reviewed by study team members and adapted for the low-literacy, low-socioeconomic status population. We selected recipes based on familiarity and ease of access to ingredients required, cultural relevancy, time required to prepare and cook, and integrability of didactic components. An RD with specialty training in culinary medicine (MAL) facilitated the eCM online monthly classes in English and Spanish. Each class lasted approximately 2 h. The study team scheduled and coordinated eCM classes. Participants randomized to eCM received a Pantry Pack, which included common pantry items used in our recipes (e.g., white vinegar, olive oil, turmeric, garlic powder). Additionally, participants received a $10 grocery gift card ahead of each class to purchase necessary ingredients.

### 2.8. Initial Study Launch Review

After the study launch (November 2021), weekly team meetings to monitor progress identified recruitment difficulties and low initial attendance for the first English and Spanish eCM groups (0 and 2 participants, respectively). In January 2022, we paused the study to solicit team-based observational feedback based on phone interactions with patients, review of recruitment notes, enrollment protocols, class structure, and engagement methods. We invited enrolled participants from the first group to participate in an interview to solicit their feedback on study operations. We conducted semi-structured interviews (*n* = 8) to solicit feedback on study appeal, operational aspects of enrollment, and barriers to participation, followed by a rapid thematic analysis [34]. Using findings from the operational interviews (including a combination of those who attended and did not attend classes) and observed feedback from team members, we identified opportunities for improvement and implemented changes to redesign study recruitment and engagement procedures to improve study enrollment and engagement.

## 3. Results

### 3.1. Study Recruitment and Enrollment Redesign

Interview data and study team observational feedback on original elements of study design helped narrow and identify change opportunities and strategies (Table 2). Overall, participants associated the study with Parkland, knew how to contact the study team if needed, and understood the study’s purpose. Participants had positive feedback regarding the food distribution. However, they also identified transportation barriers due to the pick-up location not being on a public transit route, as well as conflicts with designated food pick-up times. One participant reported, “They told me to go every first Wednesday. The first Wednesday of each month, and the last one, and the third one. That is why… right now I have not gone because I do not have a car” (INT6). The study team also noted that many participants did not have or use email, which was a barrier to signing the electronic consent form, which was required to share information with the community organization providing food. This also prevented the distribution of study supplies needed for participation because they were designed to be delivered by the community organization.

These challenges, combined with scheduling and coordination challenges for the study team, led us to implement an opt-in model for food assistance. This change negated the need for written consent and allowed us to streamline recruitment by collecting verbal consent during the initial phone recruitment call. Newly enrolled participants were provided a curated list of food assistance resources available in their ZIP Code and were encouraged to connect with resources convenient for them as needed. Initial study participants who wished to continue seeking services were encouraged to do so with our community organization directly or were provided with the same food assistance list as part of our transition plan. Since our community organization was no longer a centralized location for study onboarding, we moved onboarding procedures for eCM participants to the clinic where they received their primary care. Given the positive interview feedback about MNT, fewer changes were suggested and implemented for the MNT comparison arm aside from picking up their cooking supplies from the RDs at their clinic instead of the community organization.

### 3.2. eCM Engagement and Redesign

Participants appreciated and reinforced the importance of reminders for eCM classes. Common reasons for non-attendance included family emergencies and internet connectivity issues. eCM participants highlighted a lack of familiarity with culinary medicine classes and suggested that the opportunity to invite and cook with peers and/or family members would make them feel more comfortable. Similarly, some expressed a preference to observe rather than cook. One participant suggested using a more familiar virtual platform instead of Zoom. Additional feedback and change opportunities are shown in Table 2.

We made several changes to eCM study processes, as shown in Table 2. First, to increase familiarity and comfort with the eCM classes, we relaxed attendance policies and encouraged participants to invite family members and friends to cook alongside them during the class. We also allowed participants to observe the class if they were not ready to cook. Built on participant familiarity with cooking shows, the instructors heightened their “chef” and “sous chef” personas to enhance class connections and reduce anxiety. Between classes, the sous chef administered a 1:1 phone call with each participant to check in, review the recipe for the upcoming class, and confirm receipt of the grocery stipend. Second, to guide and standardize the Orientation meeting, we developed an eCM Orientation Checklist that included an overview of the individual benefits of eCM, a review of class recipes and grocery planning materials, as well as expectations for the first class. The Orientation meetings lasted about 20 min. We included a technical support module, including assistance with Zoom installation and tutorials on their personal device using teach-back methods [35]. If internet connectivity was identified as a potential barrier, we provided information on free hotspot rental programs at local libraries.

### 3.3. Trial Recruitment Results

**Figure 1:**
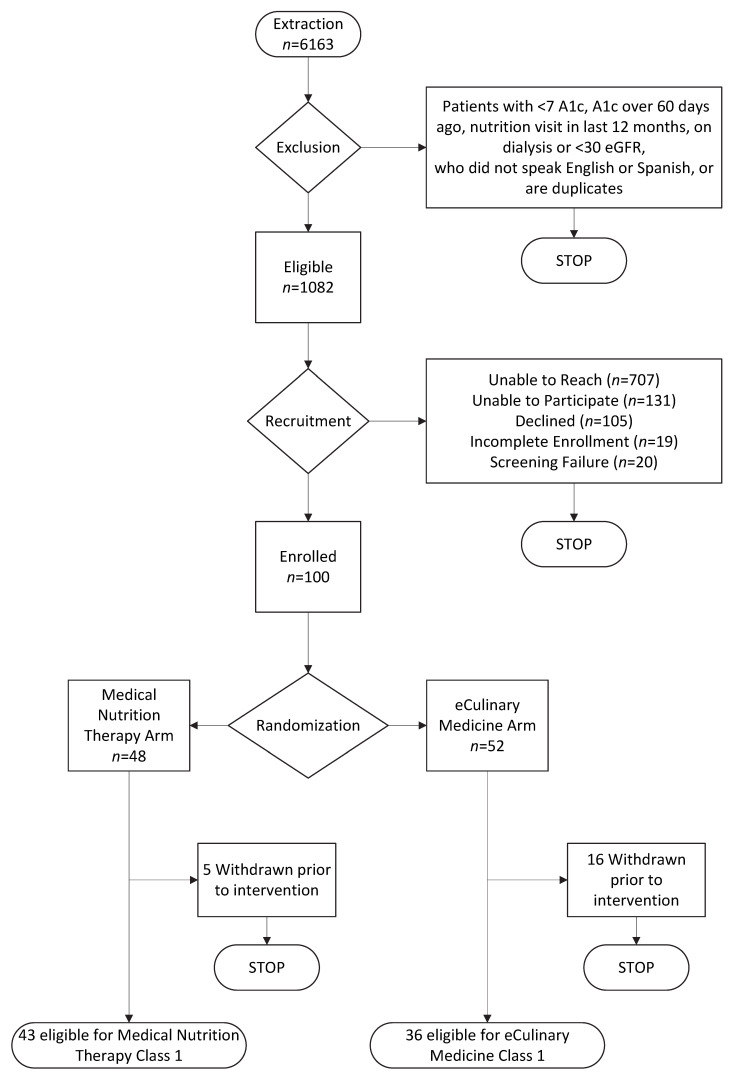
Recruitment Flow Diagram, November 2021–February 2023.

Recruitment occurred during three periods: November–December 2021, May–June 2022, and January–February 2023. The study team applied exclusion criteria during each recruitment period, as shown in Figure 1, with 1082 patients eligible during the rolling recruitment periods. Eligible patients were imported into REDCap and mailed a study invitation letter outlining the risks and benefits of the study, followed by a phone call from the study team. Most patients were unable to be reached after three call attempts (*n* = 707, 65%). Among those patients who answered, common reasons for declining participation were limited internet access or scheduling limitations (*n* = 131), not interested (*n* = 105), or failure to complete baseline enrollment procedures (*n* = 39). A total of 100 patients provided verbal consent to participate in the trial and completed the baseline survey over the phone (Figure 1).

### 3.4. Randomization and Baseline Characteristics

Participants providing verbal consent and completing the baseline survey were randomized 1:1 to a study arm (48 MNT and 52 eCM). Twenty-one (5 MNT and 16 eCM) were unable to be reached following randomization or did not pick up study supplies and were withdrawn by the study team prior to the first class, leaving 43 MNT participants and 36 eCM participants. The baseline characteristics of the 79 enrolled participants are shown in Table 3. Participants were 82% female with a mean age of 50 years (range 24–82 years). Most preferred Spanish language (56%), and nearly 40% had less than a high school education. The average household size was four persons (range 1–12), including children. Over 40% of participants reported food insecurity in the past 30 days, and 63% reported using at least one food assistance program at baseline. The average HbA1c value at baseline was 9.4% (range 7.0–15.1%). Baseline characteristics are similar between the study arms, although food insecurity was slightly higher in eCM compared to MNT (50% vs. 37%, respectively). Less than high school education was also slightly more common among the MNT participants compared to the eCM participants (42% vs. 33%).

### 3.5. Participant Engagement

**Figure 2:**
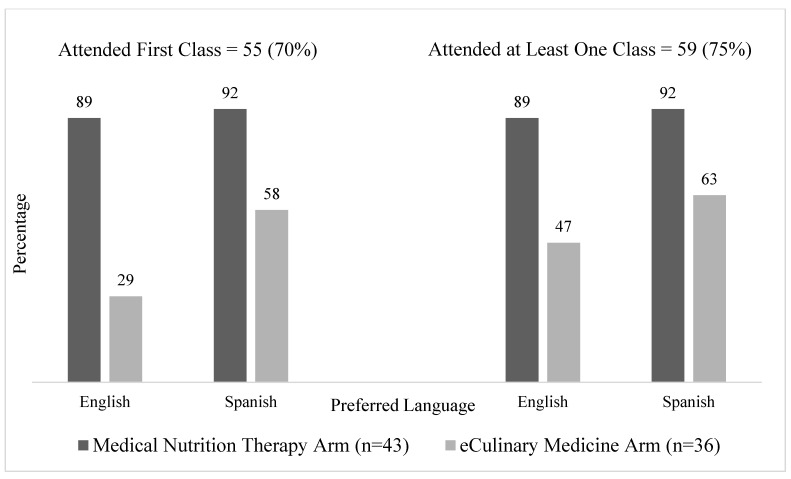
Participant Class Engagement Metrics After Re-Design by Study Arm and Preferred Language (*n* = 79).

We described how many participants attended at least one class during the six-class intervention period and, more specifically, how many participants attended the first class. Overall, 75% of participants attended at least one class (59/79), and 70% of those attended the first class (55/79). Both metrics were higher in the MNT arm compared with the eCM arm (91% vs. 56% attended at least one class, respectively; 91% vs. 44% attended the first class, respectively). There were especially low rates of first-class attendance (29%) and attending at least one class (47%) among English participants randomized to eCM (Figure 2). As this trial is ongoing, full class attendance data over the six classes, as well as qualitative reasons for retention, will be presented in future reports.

## 4. Discussion

In this pragmatic trial designed to compare the effectiveness of eCM and MNT for patients with uncontrolled diabetes, we faced multiple challenges in recruiting and engaging underserved patients in virtual nutrition interventions. After pausing the study to critically evaluate operational processes and engage participants, study team members, and other key stakeholders, we identified barriers and facilitators to study recruitment and engagement and generated actionable opportunities for process redesign [36]. Implementation of targeted changes in study procedures improved recruitment and engagement in this ongoing study.

Although we leveraged our prior experience with the design and delivery of in-person culinary medicine to account for the challenges of executing a virtual intervention in an underserved study population, we encountered technological and logistical barriers to recruitment and engagement. Most patients in our safety net health system have smartphones with video capabilities. However, many eligible participants lacked internet access. Moreover, among those with internet access who enrolled, many lacked a reliable connection capable of supporting the video platform. We did have some success connecting patients with local libraries for hotspot rentals to improve connectivity. Although our initial design provided detailed phone-based guidance on the virtual platform, many patients struggled to access and use the virtual platform to join the class. We were able to help address this barrier by conducting in-person, 1:1 technology tutorials for participants to download, install, and practice using the eCM virtual platform. Additionally, a study team member remained available on standby during the eCM classes to provide support for any unexpected technological difficulties. Although virtual interventions and education programs may help address transportation and childcare barriers [37], some participants voiced concerns about others being able to see their kitchen or distractions in their home and strength of connectivity presented challenges to joining. Providing tablets with pre-installed software may improve the engagement of underserved populations in future studies.

Although we described the culinary medicine intervention as ‘cooking classes’ to participants, participants remained uncertain about what to expect, which was a potential barrier to recruitment and engagement. While some participants were excited by the cooking classes, others were hesitant because they did not feel confident in their cooking or felt comfortable cooking along with the instructors. These findings are similar to other studies suggesting that a lack of interest or knowledge about cooking creates uncertainty and may pose a barrier to participating in cooking classes [12,38]. Redesign strategies targeting these barriers included developing the persona of the chef, adding a ‘sous chef’ to increase comfort and continuity between classes, and allowing participants to observe the first class or invite a friend or family member to assist in enhanced engagement. Engaging family members, especially children, is widely accepted in other programs [18,19,39]. Aside from these modifiable strategies, participants also indicated that personal and life circumstances frequently interfered with participation, such as sudden changes to work schedules, family emergencies, and personal health situations that prevented them from joining. As shown earlier in Table 2, we were not able to implement all the suggestions provided by participants to improve engagement in our current study (Table 2). These included video testimonials from peers who have participated in the classes and the use of pictures of ingredients in recipes to address nutrition and food literacy.

Preliminary findings from this redesign indicate that MNT participants had higher engagement at the first class than eCM participants even after changes were implemented; however, participants who joined the first eCM class were more likely to return, especially Spanish speakers. Notably, the eCM intervention was not directly affiliated with the health system, and classes were held in the evenings, which may play a role in eCM’s lower engagement. The MNT intervention, which was delivered using health system RDs at the patient’s primary care clinic during business hours, was more familiar to participants and likely viewed as part of their healthcare. The RDs conducted their own scheduling, reminder, and outreach calls in the case of “no show” appointments, which led to a strong rapport with study participants. Such patient-dietitian relationships are key components of successful dietary interventions; making sessions feel personal and individualized, even when delivered in a group setting, is important for building interest and engagement [40,41]. Culinary medicine interventions conducted within familiar local infrastructure, such as the health system where a patient is already receiving care, a food pantry, or other community organizations where a client has already established a trusting relationship, or programs linked more tightly to clinical care may increase engagement in future studies [38,39,42,43].

Although we initially planned to distribute cooking utensils, recipe binders, and pantry staples at the community organization, low show rates for food pick-up resulted in eCM participants not having the necessary items for their class. This resulted in the differential withdrawal of participants from the eCM arm relative to the MNT arm, which did not require this additional step. Participants identified transportation, lack of familiarity with the community organization, and conflicts with food pick-up times as key barriers. Our original study design was ‘opt out’ for food assistance in collaboration with our community organization. This required a signed informed consent and HIPAA authorization to share participant information with our community organization, which posed a substantial barrier to recruitment via telephone. Given this and the participant barriers to food pick-up, we transitioned to an ‘opt in’ food distribution model, which did not require written consent and gave participants greater choice and flexibility in selecting their food resource. This model also allowed us to move the in-person eCM Orientation meeting to the participant’s primary care clinic, which was familiar and more accessible. We explored the possibility of delivering supplies directly to their home, but we did not have sufficient funding or staffing to support this. Home delivery of supplies and groceries may limit the need for shopping and help alleviate barriers to participation in virtual culinary medicine classes where participants cook in their home kitchens.

## 5. Conclusions

Recruitment and engagement of underserved populations in virtual culinary medicine and nutrition studies is challenging. Although our study team had substantial experience in the delivery of in-person culinary medicine and nutrition interventions, translating these experiences to virtual platforms presented an array of technological barriers and logistical challenges. Our study is unique in that we enrolled participants in a randomized trial of culinary medicine rather than evaluating outcomes in participants who self-select into such a program. Virtual culinary medicine and nutrition trials require significant infrastructure and support from study teams to facilitate participation in interventions. By engaging study participants, study team members, and community partners, we identified actionable items to improve study processes to successfully recruit and engage participants in both eCM and virtual MNT.

## Figures and Tables

**Table 1:** Comparison of Study Arms with respect to the Role of the Facilitator, Setting, Requirements, Content, and Educational Approach.

Concept	eCulinary Medicine	Medical Nutrition Therapy
Facilitator Background	Lead Bilingual RDcm (Contractor), Assistant Bilingual facilitator with CM-specific training or RDcm	English-speaking RD Parkland provider, Spanish-speaking RD Parkland provider
Educational Approach	Experiential learning in a small, virtual group setting focused on practical meal planning and preparation, culinary skills building, and food presentation and discussion. Participants were given binders with recipes and handouts, and both a practical, “hands-on” cooking experience and a didactic lesson from the facilitator reinforced themes of a nourishing dietary pattern for health promotion.	Traditional diabetes education model tailored for patients with known diabetes. The session content generally followed a predetermined outline of six sessions but was adjusted for individual patient needs based on ADA standards of care nutrition consensus statements for diabetes and prediabetes.
Frequency	Sessions were held on a fixed monthly schedule on Tuesday evenings, with English one week and Spanish the following week.	Sessions were held monthly and scheduled during designated appointment slots based on RD’s schedule during their working hours.
Scheduling	Participants were given a class schedule at the beginning of the intervention and reminder messages prior to each class. All contact and attendance documentation occurred outside of the health record and their established healthcare practice.	Participants were scheduled per clinic workflow and by appointment. RD called the participants to schedule an appointment. If the participant “no-showed”, RDs reschedule. All contact and attendance documentation occurred in the electronic health record.
Setting/Timing	All participants simultaneously joined the 2-h class from their individual kitchens via Zoom with phone/device camera and microphones turned on. A study team member managed the Zoom chat and session controls. The facilitator had one camera on his face, and another on the demonstrative cutting board for the live demo.	1:1 sessions with the language-specific RD lasted 45–60 min and were virtual or in-person in the RD clinic-based office, per patient preference. Language-specific group sessions lasted 60–90 min and took place in a conference room at the clinic. Virtual sessions took place via phone call.
Session Handouts and Educational Visuals	Recipes and educational handouts were selected based on the cultural representation of learners from the Diabetes and Carbohydrate Module of the Health Meets Food curriculum [33].	Food models, measuring cups, various nutrition handouts, fact sheet handouts, sugar demo posters, exercise videos, and food labels were provided by the RDs.
Resources Issued to Subjects	Cooking equipment (frying pan, cutting board, measuring cups, cutlery), Recipe binders, grocery shopping lists, Zoom instructions, Pantry Pack (non-perishable foods, oils, and spices), Food Assistance Resource List, $10 Grocery stipend per class	Cooking equipment (frying pan, cutting board, measuring cups, cutlery)
Pre-Class Preparation	Participants were expected to review the shopping list and procure ingredients before the start of each class.	Participants were expected to find transportation to and from the clinic for the in-person meetings.
Session Focus	eCulinary Medicine	Medical Nutrition Therapy
Session 1	Knife safety demonstration; kitchen orientation, temperature safety, and “danger zone” including cross-contamination; How to make your own vegetable stock and salad dressing; Common kitchen tools, common cooking abbreviations, appropriate knife cuts, and cooking terms; How to cut a yellow onion, a bell pepper.	1:1 Virtual/In-Person Meeting: Medication adherence related to meal timing; Glucose monitoring and pattern management intro; Diet history review; Assess barriers; Make referrals; Carbohydrate awareness; Beverage recommendations (including alcohol); Discuss recent lab results; Exercise recommendations; Goal Setting
Session 2	Caramelization of carbohydrates for flavor-building, balancing texture for palatability; Tips for cooking with whole grains; Alternative use and storage of leftovers.	1:1 Virtual/In-Person Meeting: Meal composition and timing relative to medication and glucose log; Review carbohydrate awareness and consistency; Snack recommendations and options
Session 3	Meal planning tips; Substitutions for oils and butters in baking and other dishes; Tips for shopping for and preparing/storing seafood/shellfish for best taste and safe handling.	Group In-Person Meeting: Meal planning tips; Sugar-containing beverages; fat content impact on Diabetes and Cardiovascular disease; Plate method principles with demonstration and participation; Food label review; Grocery shopping and cooking tips; exercise adherence tips
Session 4	Discuss sofrito and flavor building, mirepoix, especially in vegan/vegetarian cooking, and strategies for flavor building when reducing red meat and saturated fat.	1:1 Virtual/In-Person Meeting: Review food label reading using items from pantry/fridge
Session 5	Review previous sessions, assess knowledge gaps, and address any deficits; Tips for adding more vegetables and whole grains into familiar dishes.	Group In-Person Meeting: Importance of weight loss/maintenance; Eating and preparing meals as a family; Heart Health- increasing fibers and healthy fats; Maintaining motivation and how to get back on track; Problem-solving when eating outside of the home, holidays, etc.
Session 6	Tips for when to use which type of fat/oil; Education on smoke point.	1:1 Virtual/In-Person Meeting: Review goals and progress

**Table 2:** Original Design with Stakeholder Feedback and Description of Changes.

Original Recruitment Design	Interview/Observational Feedback	Description of Change
Study team schedules participants for pick up, conducts reminder calls, and coordinates with DBC * staff to meet participants during first DBC food distribution to complete baseline consent/survey	*Study Staff Feedback* ○ Participants who miss DBC food pick-up cannot sign consent and do not pick up supplies (eCM arm). ○ Participants cannot carry the full two-week supply of food distribution at DBC to the bus stop. ○ Scheduling and coordinating food distribution pick-ups at DBC is cumbersome for the study team and distracts from primary recruitment tasks.	Switch to a well-known, more accessible location (their clinic) for participants to meet with study staff. Change to phone-based opt-in for food assistance picks up at community organizations rather than an opt-out model. Allow participants who wish to continue going to DBC to pick up on a weekly basis to scale down the amount of food they must carry. Work with food assistance leaders (Crossroads, Sharing Life, and DBC) to provide a Food Assistance Resource List specific to ZIP code to decentralize efforts and allow participants to choose resources closer to them.
	*Participant Feedback* ○ DBC location is inconvenient, and participants expressed unfamiliarity/uncertainty with the process of using services.	
Study team contacts patients to notify them of the study opportunity and randomization arm	*Participant Feedback* ○ Participants who attend classes are highly motivated by individual benefits and find a way to make it happen	Emphasize individual benefits of the class with enrolled participants during the notification calls and reiterate during in-person Orientation meetings (eCM only).
Study team asks patients about internet access prior to consent as a screening question	*Study Staff Feedback* ○ Lack of internet-connected devices leading to exclusion of many patients	Target recruitment to those with email addresses or MyChart access as a proxy for internet connectivity ^.
Original Medical Nutrition Therapy	Interview/Observational Feedback	Description of Change
Study recruitment is completed by the study staff, and participants are transitioned into the MNT program	*Study Staff Feedback* ○ Participants asking MNT staff about food pick up or asking study-specific questions	Meet periodically with RDs to ensure they are aware of study procedures and how to handle study-related questions. Introduce MNT staff RDs to DBC staff and set up a facility tour to familiarize them with local food assistance resources.
MNT program run and coordinated by clinic-based RDs with experience in diabetes management within context of a clinic visit	*Participant Feedback* ○ High level of familiarity and relationship development with nutritionist, dedicated staff with recognizable interaction style (“patient visit”) ○ Easier for participants to join due to flexibility (WebEx link via text, 1:1 scheduling, in-person, or virtual, unlimited reminders), but limited to working hours	Good feedback from participants, which did not require any redesign. Use this visit model to frame the redesign changes for the culinary medicine arm, including permanent instructor staffing, consistent/familiar relationship building with participants, ability to schedule make-up sessions (i.e., rescheduling), participant communication via text message, and texting the link to the class sessions.
Original Culinary Medicine Design	Interview/Observational Feedback	Description of Change
Participants procure their ingredients and log in to join eCM class on Zoom from their own kitchen	*Participant Feedback* ○ Not ready to cook when joining the class and prefer to “watch” video rather than cook in class. ○ Would prefer to cook with their family members or friends in the class. ○ Concerns about cooking at home and possible distractions (kids, pets, messy, etc.) ○ Confusion about what to expect (missed pick up of supplies), what culinary medicine is, why, and how they should join. ○ Unfamiliar with teacher/classmates, class format, timid about joining the class. ○ Internet-connected device they have available to them is too small to see a video screen; or their internet service and/or connection is not strong enough for videoconferencing.	Add a “watch only” option for the first class for those who are not ready to cook and open classes to peer and family attendance. Send video testimonials from other diabetes patients encouraging participants to do the classes ^. Revise info sheet and call scripts for clear and consistent messaging about the study goals, benefits, and expectations. Meet each participant in-person at a familiar location (i.e., their clinic) to conduct in-depth eCM Orientation meetings. Develop eCM Orientation Checklist to assess comfort level, identify household barriers, establish rapport and importance of class attendance, emphasize individual benefits and socialization, discuss comfort level with the first recipe (Tacos), and deliver a “tech module” for personalized app installation and teach-back learning of Zoom platform. Develop a Chef Biography and persona that participants can get excited to cook with and can relate to. Select “Sous Chefs” to assist with class and model desired behavior for participants, for example, asking questions, making mistakes, having messy kitchens, etc. Purchase participant tablets to ensure everyone has a large viewing screen for class participation ^. Share public library resource info sheet for internet hotspot rental program.
Participants receive a call from the study team the day prior to the class to remind them of the class time and what recipe will be prepared	*Study Staff Feedback* ○ Initial class attendance low. Participants experience events day-to-day that prevent them from joining the class, even when the day before, they voiced that they were planning to join. ○ Time in between monthly classes is vast, and a lot can happen. ○ Personal touch is important to participants before, during, and after intervention.	Add personalized “check-in” calls in between classes to ask how they are doing, reiterate class material, see if they have attempted the recipe again, encourage them to submit a photo of the meal to share with the class, and ensure they are prepared for the upcoming class. Use this opportunity to discuss food preferences and substitutions for upcoming classes on a 1:1 basis outside of the group setting. Text the direct link with more engaging messaging: “Class starts in 2 h! Let us get cooking!—Chef Miguel”. Use WhatsApp or WebEx as more familiar platforms ^. Include a copy of the shopping list for the next class along with the grocery stipend they receive by mail to make sure they are prepared to cook and know what we are making.
Participants receive recipe binders with cooking instructions, handouts, and shopping lists to prepare for each class	*Study Staff Feedback* ○ The written material presents a food literacy concern for participants, especially Spanish speakers, who are unfamiliar with recipes, cooking utensils, terminology.	Contract bilingual, culturally competent RD for consistency across English and Spanish groups and for the duration of the class (6 months). Provide pictures of the finished meal in addition to the step-by-step recipes and ingredients list ^. Use in-person Orientation meetings to review each ingredient in the shopping list together with the participants in their native language to identify what they already have at home vs. what they need to buy before the class. Discuss their baseline understanding of the first recipe and potential obstacles to joining the class or preparing the ingredients (e.g., allergies, cheese affordability and preferences, etc.).

* DBC = Dallas Bethlehem Center, our community-based partner for food pantry distribution. ^ Concepts that were suggested by stakeholders but were not implemented.

**Table 3:** Participant Characteristics at Baseline by Study Arm (*n* = 79).

	Total *n* = 79	Medical Nutrition Therapy Arm *n* = 43	eCulinary Medicine Arm *n* = 36
Age (*n*, %)	49.7, SD 11.26, range: 24–82	48.7	50.9
20–29	4 (5.1%)	2 (4.7%)	2 (5.6%)
30–39	12 (15.2%)	9 (20.9%)	3 (8.3%)
40–49	21 (26.6%)	10 (23.3%)	11 (30.6%)
50–59	29 (36.7%)	15 (34.9%)	14 (38.9%)
60–69	11 (13.9%)	6 (14.0%)	5 (13.9%)
70–79	1 (1.3%)	1 (2.3%)	0 (0.0%)
80–89	1 (1.3%)	0 (0.0%)	1 (2.8%)
Female (*n*, %)	65 (82%)	35 (81%)	30 (83%)
Preferred language Spanish (*n*, %)	44 (56%)	25 (58%)	19 (53%)
Less than high school education (*n*, %) *	30 (38%)	18 (42%)	12 (33%)
Food Insecure (*n*, %) *	34 (43%)	16 (37%)	18 (50%)
Using Food Assistance (*n*, %) *	50 (63%)	27 (63%)	23 (64%)
Household Size (average number of people, including children) *	3.8, SD 2.06, range: 1–12	4.1	3.6
Baseline HbA1c Value (average mg/dL)	9.4%, SD 1.84, range: 7.0–15.1%	9.6%	9.3%

* Self-Reported during baseline survey.

## Data Availability

The data are not publicly available due to the ongoing status of the study which is not de-identified at this time.
